# Aggressive surgical approach with vascular resection and reconstruction for retroperitoneal sarcomas: a systematic review

**DOI:** 10.1186/s12893-023-02178-1

**Published:** 2023-09-12

**Authors:** Hankui Hu, Qiang Guo, Jichun Zhao, Bin Huang, Xiaojiong Du

**Affiliations:** Division of Vascular Surgery, Department of General Surgery, West China Hospital, Sichuan University, Chengdu, 610041 Sichuan Province China

**Keywords:** Retroperitoneal sarcoma, Surgery, Vascular resection, Overall survival, Systematic review

## Abstract

**Background and aim:**

Surgery is the mainstay of treatment and completeness of surgical resection is critical to achieve local control for retroperitoneal sarcoma (RPS). En-bloc resection of adjacent organs, including major abdominal vessels, is often required to achieve negative margins. The aim of this review was to summarise the available evidence to assess the relative benefits and disadvantages of an aggressive surgical approach with vascular resection in patients with retroperitoneal sarcoma (RPS).

**Methods:**

We searched PubMed, the Cochrane Library, and EMBASE for relevant studies published from inception up to August 1, 2022. We performed a systematic review of the available studies to assess the safety and long-term survival results of vascular resection for RPS.

**Results:**

We identified a total of 23 studies for our review. Overall postoperative in-hospital or 30-day mortality rate of patients with primary iliocaval leiomyosarcoma was 3% (11/359), and the major complication rate was 13%. The recurrence-free survival (RFS) rates after the follow-up period varied between 15% and 52%, and the 5-year overall survival (OS) rates ranged from 25 to 78%. Overall postoperative in-hospital or 30-day mortality rate of patients with RPSs receiving vascular resection was 3%, and the major complication rate was 27%. The RFS rates after the follow-up period were 18–86%, and the 5-year OS rates varied between 50% and 73%. There were no significant differences in the rates of RFS (HR: 0.97; 95% CI: 0.74–1.19; *p* = 0.945) and OS (HR: 1.01; 95% CI: 0.66–1.36; *p* = 0.774) between the extended resection group and tumour resection alone group.

**Conclusions:**

With adequate preparation and proper management, for patients with RPSs involving major vessels, aggressive surgical approach with vascular resection can achieve R0/R1 resection and improve survival.

**Supplementary Information:**

The online version contains supplementary material available at 10.1186/s12893-023-02178-1.

## Introduction

Soft tissue sarcomas (STSs) are rare malignant tumours that represent approximately 1% of all adult malignancies [1]. Approximately 15–20% of all STS arise in the retroperitoneum, with a 5-year overall survival (OS) rate in the range of 39–70% [2, 3]. Retroperitoneal sarcomas (RPSs) often progress asymptomatically and are thus only detected incidentally when the substantially enlarged tumour compresses the surrounding organs [4]. Patients presenting with back pain or abdominal distention already have a large tumour with close proximity to critical structures, such as major vessels. With respect to the treatment of RPS, the use of adjuvant radiotherapy and chemotherapy varies widely among institutions because of the lack of evidence supporting their benefit [5]. Thus, surgical resection remains the cornerstone of therapy and the only potentially curative therapy for patients with RPS [5].

Guidelines on the surgical management of RPS are still lacking and remain controversial, owing to its low incidence [6]. For example, the criteria for unresectability remains undefined, and the indication and eligibility for surgical resection vary by medical centre. Patients with residual macroscopic disease are often referred to specialised centres because the appropriateness of en-bloc resection for organs adherent to the tumour needs to be determined intraoperatively. The trans-Atlantic RPS working group recently updated the consensus on management of primary RPS in adults [7]. The update established criteria for technical non-resectability as involvement of the superior mesenteric artery, aorta, coeliac trunk, and/or portal vein; bone involvement; growth into the spinal canal; invasive extension of retrohepatic inferior vena cava leiomyosarcoma into the right atrium; and infiltration of multiple major organs and/or major vessels [7]. However, vascular reconstructions, which enable radical resection of RPSs in patients with advanced disease, have been successfully performed in many studies [8, 9]. The inferior vena cava (IVC) and iliac veins (IVs) were the most common vessels involved in RPS resection [9]. Aggressive resection with involved major blood vessels such as the IVC and IVs may improve R0 resection rates; however, the benefit of converting R1 to R0 resections is unclear, and vascular resection might be associated with an increased risk of postoperative complications [10]. Therefore, to determine the relative benefit and disadvantages of an aggressive surgical approach with vascular resection in patients with RPS, we conducted a systematic review to assess the safety and long-term survival results of vascular resection. We also conducted a meta-analysis to compare the clinical outcomes between vascular resection and tumour resection alone in patients with RPS.

## Methods

### Search strategy

For this systematic review, we conducted a search in MEDLINE, Embase, and Web of Science (inception to August 1, 2022). A comprehensive search was performed with the following terms: ‘retroperitoneal tumour,’ ‘retroperitoneal neoplasm,’ ‘retroperitoneal sarcoma,’ and ‘vascular’ or ‘inferior vena cava’ or ‘iliocaval’ (specific search strategies are listed in Supplementary Text 1). The inclusion criteria were as follows: original articles in English reported or accepted in a peer-reviewed journal, and studies that included participants who underwent vascular resections for RPS (primary or recurrent). We reviewed the reference lists of the included papers. We excluded case reports or case series with a participant sample size below 10. We also excluded studies reported only as meeting abstracts and unpublished studies, and those that did not provide hazard ratios (HRs) or confidence intervals (CIs). This systematic review and meta-analysis was conducted according to the Preferred Reporting Items for Systematic Reviews and Meta-analyses (PRISMA) guidelines for systematic reviews (Supplementary Table 1) [11].

### Data extraction and quality assessment

Two of the authors independently screened the titles and abstracts, reviewed the full texts, extracted the data, and assessed the risk of bias. The methodological quality of case-control and cohort studies was assessed by two authors independently by using the Newcastle-Ottawa Scale, which assigns 4 points for selection, 2 points for comparability, and 3 points for outcome [12]. A high score of an assessed study corresponds to a high quality. We collected the study characteristics (name of the first author, publishing year, sample size, follow-up period), tumour characteristics (histological subtype, French Federation of Cancer Centers Sarcoma Group (FNCLCC) grade [13], and tumour status), surgical characteristics (margin status and vascular reconstruction), and outcomes (OS, recurrence-free survival (RFS), postoperative complications, and 30-day mortality). Postoperative complications were scored by the Clavien-Dindo grading system with grade III or greater considered as severe complications [14].

### Statistical analysis

All outcomes were dichotomous data. Heterogeneity was assessed using the I^2^ statistic, with I^2^ values of 25%, 50%, and 75% considered to indicate low, moderate, and high heterogeneity, respectively. The primary outcome was OS. The secondary outcomes were RFS, postoperative complications and early postoperative mortality. Pooled HRs and 95% CIs were estimated to compare the risk of recurrence or OS. Pooled odds ratios (ORs) with 95% CIs were estimated to compare the risk of postoperative complication or early postoperative mortality between an aggressive surgical approach with vascular resection and tumour resection alone. For time-to-event outcomes, including RFS and OS, HRs and their associated variances were extracted, or estimates were calculated when possible, using the methods described by Tierney et al. [15]. Publication bias was assessed using funnel plots. All statistical analyses were performed using Stata/MP, version 16.0 (StataCorp LLC). All tests were two sided, and *P* < 0.05 was considered statistically significant.

## Results

### Study characteristics

**Fig. 1:**
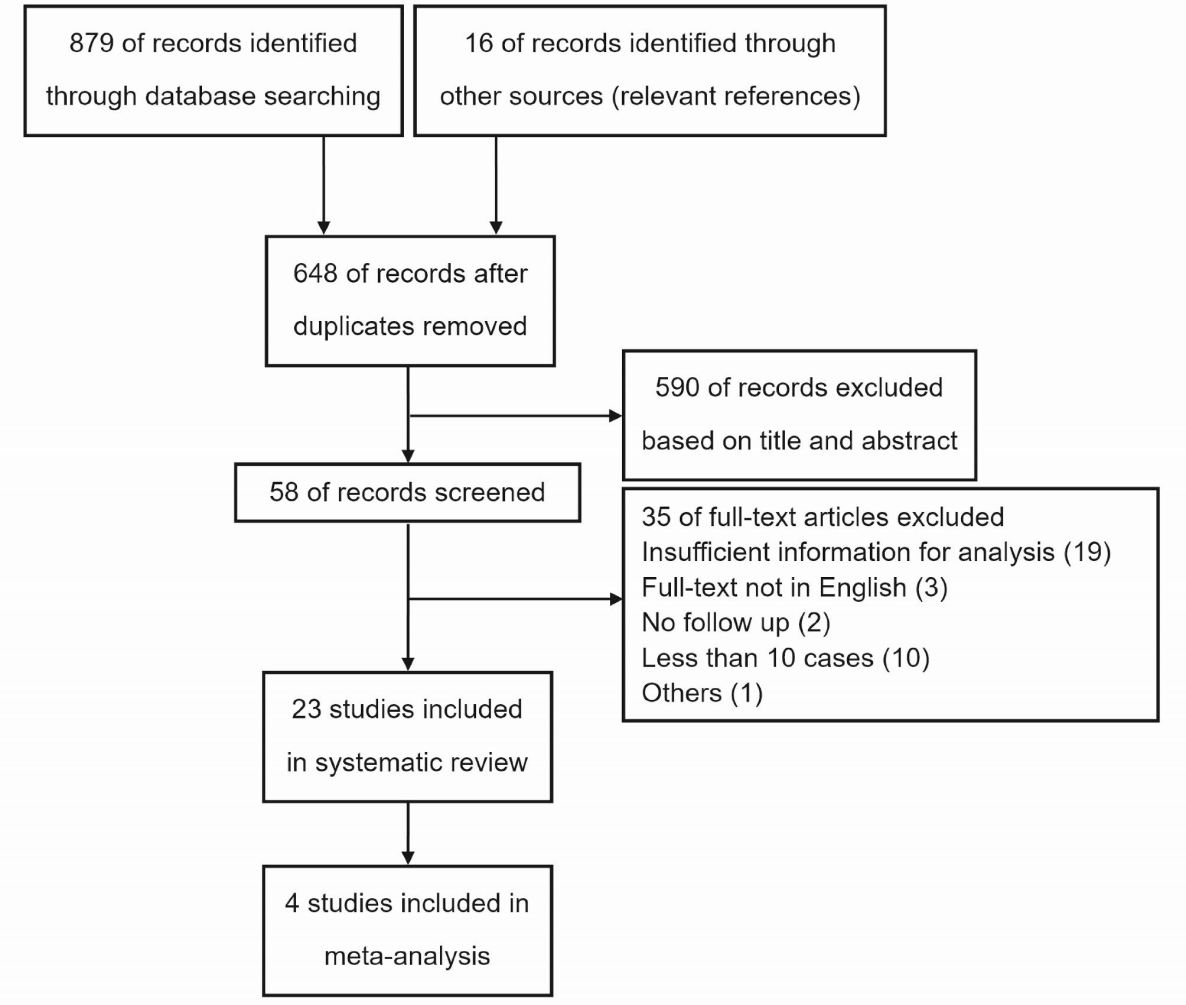
PRISMA Flow diagram

Of the 648 citations identified, we selected 58 potentially relevant abstracts for detailed assessment. Twenty-one studies met our inclusion criteria. Characteristics of the included studies are shown in Table 1. From the 23 studies included, there were 4 cohort studies and 19 observational studies describing 699 patients [10, 16–37]. The PRISMA flow diagram, showing the entire review process from the original search to the final selection of studies, is presented in Fig. 1. The sample size of the studies varied between 11 and 120 participants. The follow-up duration varied between the studies. In general, most of the RPSs were primary (93%) and underwent total gross excision (92%). The inferior vena cava (IVC) was the most involved major vessel. In total, 12 studies [16–20, 22, 24, 26–28, 35, 36] (359 participants) reported data on primary iliocaval leiomyosarcoma, 11 studies [10, 21, 23, 25, 29–34, 37] (340 participants) reported data on other RPSs undergoing vascular resection. The predominant histological subtype was leiomyosarcoma (55%, 144/253) followed by liposarcoma (30%, 76/253). Most tumors were classified as high grade G3 (41%, 89/217). Four studies [10, 29, 31, 32] (959 participants) compared the long-term outcomes between aggressive surgical approach with vascular resection and tumour resection alone in patients with RPS. Characteristics of the included 4 studies are shown in Table 2. Two of the four studies included for meta-analysis were propensity-matched analyses [10, 32]. Overall risk of bias in this analysis was deemed low to moderate (Supplementary Table 2).

**Table 1:** Characteristics of the studies included in the systematic review on vascular resection in patients with retroperitoneal sarcoma

Study	Patients	Histological subtype (%)	FNCLCC grade (%)	Tumour status (%)	Follow-up (months)	Vascular resection (%)	Margin status (%)	30-day mortality (%)	Complication rate (%)	RFS (%)	OS (%)
Dzsinich 1992^16^	13	Lei, 100	NA	P, 100	27	IVC 100	R0/R1 93	7	23	31	3y 38
Mingoli A1997^17^	120	Lei, 100	NA	P, 100	32	IVC 100	R0/R1 100	3	6	42	5y 37
Hines1999^18^	14	Lei, 100	G1, 14; G2, 29; G3, 51	P, 100	25	IVC 100	R0/R1 86; R2 14	0	NA	43	5y 53
Hollenbeck 2003^19^	21	Lei, 100	NA	P, 100	24	IVC 100	R0/R1 84; R2 16	10	19	33	5y 33
Kieffer 2006^20^	20	Lei, 100	NA	P, 100	44	IVC 100	NA	20	NA	15	5y 35
Schwarzbach 2006^21^	25	Lip, 16; Lei, 48; MFH, 8	G1, 8; G2, 20; G3, 68	P, 64; R, 36	19	IVC 48 IV 16 AA 24 IA 12	R0/R1 68; R2 32	4	36	18	2y 56
Ito 2007^22^	20	Lei, 100	G1, 10; G2, 35; G3, 50	P, 100	47	IVC 100	R0/R1 95; R2 5	0	NA	30	5y 62
Fiore 2012^23^	15	Lip, 7; Lei, 80	G1, 20; G2, 27; G3, 53	P, 100	32	IVC 100	R0/R1 100	0	7	67	3y 80
Mann 2012^24^	17	Lei, 100	G1, 6; G2, 47; G3, 47	P, 82; R, 18	49	IVC 100	R0/R1 100	0	35	41	5y 56
Bertrand 2016^25^	22	Lip, 54; Lei, 32	G1, 6; G2, 42; G3, 42	P, 65; R, 35	34	IVC 59 IV 41 IA 36 AA 9	R0/R1 100	0	18	NA	3y 61
Cananzi 2016^26^	11	Lei, 100	G1, 27; G2, 27; G3, 36	P, 100	93	IVC 100	R0/R1 91; R2 9	0	9	36	5y 78
Illuminati 2016^27^	27	Lei, 100	NA	P, 100	60	IVC 67 IV 33	R0/R1 100	0	22	33	5y 54
Roland 2016^28^	42	Lei, 100	G1, 11; G2, 26; G3, 63	P, 100	60	IVC 100	R0/R1 80; R2 20	0	NA	35	5y 65
Tan 2016^29^	67	NA	NA	NA	NA	NA	NA	NA	NA	NA	NA
Wortmann 2016^30^	20	NA	NA	P, 64; R, 36	24	IV 10 IA 70 AA 25	NA	5	35	33	2y 69
Ikoma 2017^31^	36	Lei, 100	NA	NA	NA	IVC 100	R0/R1 100	NA	NA	NA	NA
Blair 2018^32^	32	Lip, 19; Lei, 81	G1, 44; G2, 47; G3, 9	P, 84; R, 16	37	IVC 100	R0/R1 91; R2 9	0	16	25	5y 50
Ferraris 2019^33^	67	Lip, 25; Lei, 58	G1, 10; G2, 43; G3, 46	P, 93; R, 7	58	IVC 64 IV 42 AA 6 IA 19	R0/R1 97; R2 3	3	22	40	5y 56
Homsy 2020^34^	17	Lip, 29; Lei, 59	G1, 0; G2, 35; G3, 59	P, 94; R, 6	27	IVC 53 IV 24 AA 35 IA 18	R0/R1 68; R2 32	0	29	47	3y 80
Ong 2020^35^	30	Lei, 100	G1, 13; G2, 63; G3, 23	P, 100	70	IVC 67 IV 33 IA 20	R0/R1 97; R2 3	0	17	43	5y 32
Spolverato 2021^10^	24	Lip, 100	G1, 25; G2, 54; G3, 21	P, 100	38	IVC 38 IV 50 IA 29	R0/R1 96; R2 4	0	54	52	5y 60
Goel 2022^36^	24	Lei, 100	NA	P, 100	25	IVC 100	R0/R1 100	4	4	38	5y 25
Li 2022^37^	15	Lip, 53; Lei, 13	NA	P, 60; R, 40	21	IVC 20 IA 100	R0/R1 100	0	13	86	5y 73

Lip, Liposarcoma; Lei, Leiomyosarcoma; MFH, Malignant fibrous histiocytoma; P, Primary; R, Recurrent; NA, data not available; R status, Resection status; IVC, Inferior vena cava; IV, Iliac vein; IA, Iliac artery; AA, Abdominal aorta

**Table 2:** Characteristics of the studies comparing vascular resection group and tumour resection alone group

Study	Patients (n)		Tumor size (cm)		Histological subtype (%)		FNCLCC grade (%)		Tumour status (%)		Margin status (%)	
	V	T	V	T	V	T	V	T	V	T	V	T
Tan 2016^29^	67	608	NA	NA	NA	NA	NA	NA	NA	NA	NA	NA
Ikoma 2017^31^	36	136	NA	NA	Lei, 100	Lei, 100	NA	NA	NA	NA	R0/R1 100	R0/R1 100
Blair 2018^32^	32	96	11.3	10.2	Lip, 19; Lei, 81	Lip, 19; Lei, 81	G1, 6; G2,31; G3, 63	G1, 12; G2, 28; G3, 60	P, 84; R, 16	P, 78; R, 22	R0/R1 93; R2 9	R0/R1 88; R2 12
Spolverato 2021^10^	24	401	25	25	Lip, 100	Lip, 100	G1, 25; G2/G3, 75	G1, 33; G2/G3, 67	P, 100	P, 100	R0/R1 96; R2 4	R0/R1 98; R2 2

V, vascular resection group; T, tumour resection alone group; Lip, Liposarcoma; Lei, Leiomyosarcoma; P, Primary; R, Recurrent; NA, data not available; R status, Resection status

### Reported outcomes for vascular resection in patients with RPS

Postoperative in-hospital or 30-day mortality rate of patients with primary iliocaval leiomyosarcoma was reported by 12 studies including 359 patients (0–20%), and the overall mortality rate was 3% (11/359). Major complications were reported by 8 studies [16, 17, 19, 24, 26, 27, 35, 36], and the major complication rates were 4–54% (overall major complication rate 13%). The RFS rates after the follow-up period were 15–52%. Eleven studies reported the 5-year OS rate [17–20, 22, 24, 26–28, 35, 36], ranging from 25 to 78%.

Nine studies reported 30-day or in-hospital mortality for RPSs undergoing vascular resection to be 0–8% (overall mortality rate 3%) [10, 21, 23, 25, 30, 32–34, 37]. Nine of the 11 studies reported major complications [10, 21, 23, 25, 30, 32–34, 37], ranging from 7 to 54% (overall major complication rate 27%). Seven studies (215 patients) reported clinical outcomes of RFS rates of 18–86% [10, 21, 23, 30, 32–34, 37]. There were 4 studies (237 patients) that reported 5-year OS rates that varied between 50% and 73% [10, 32, 33, 37].

### Vascular resection versus tumour resection alone

**Fig. 2:**
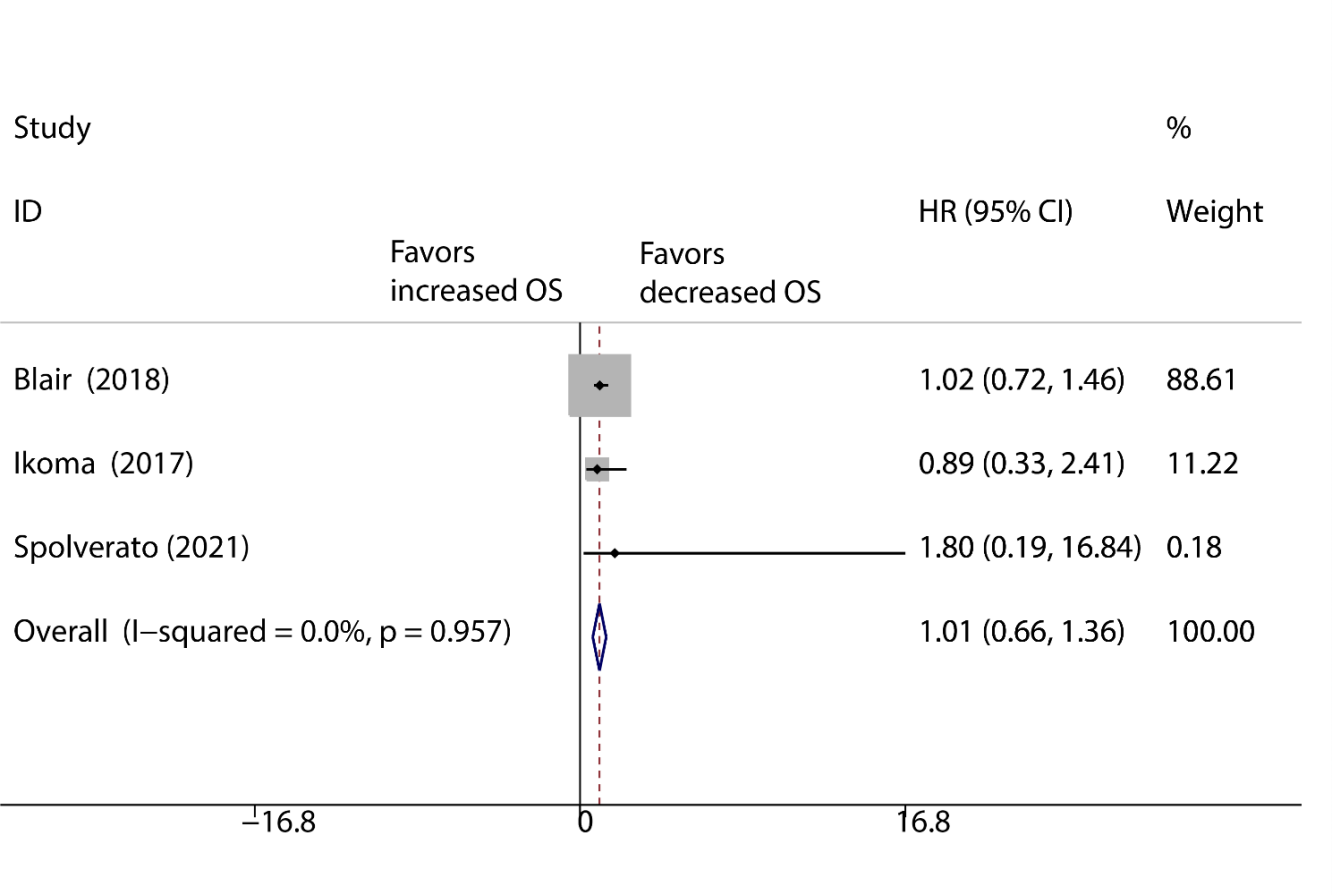
Pooled disease-free survival of vascular resection versus tumour resection alone

**Fig. 3:**
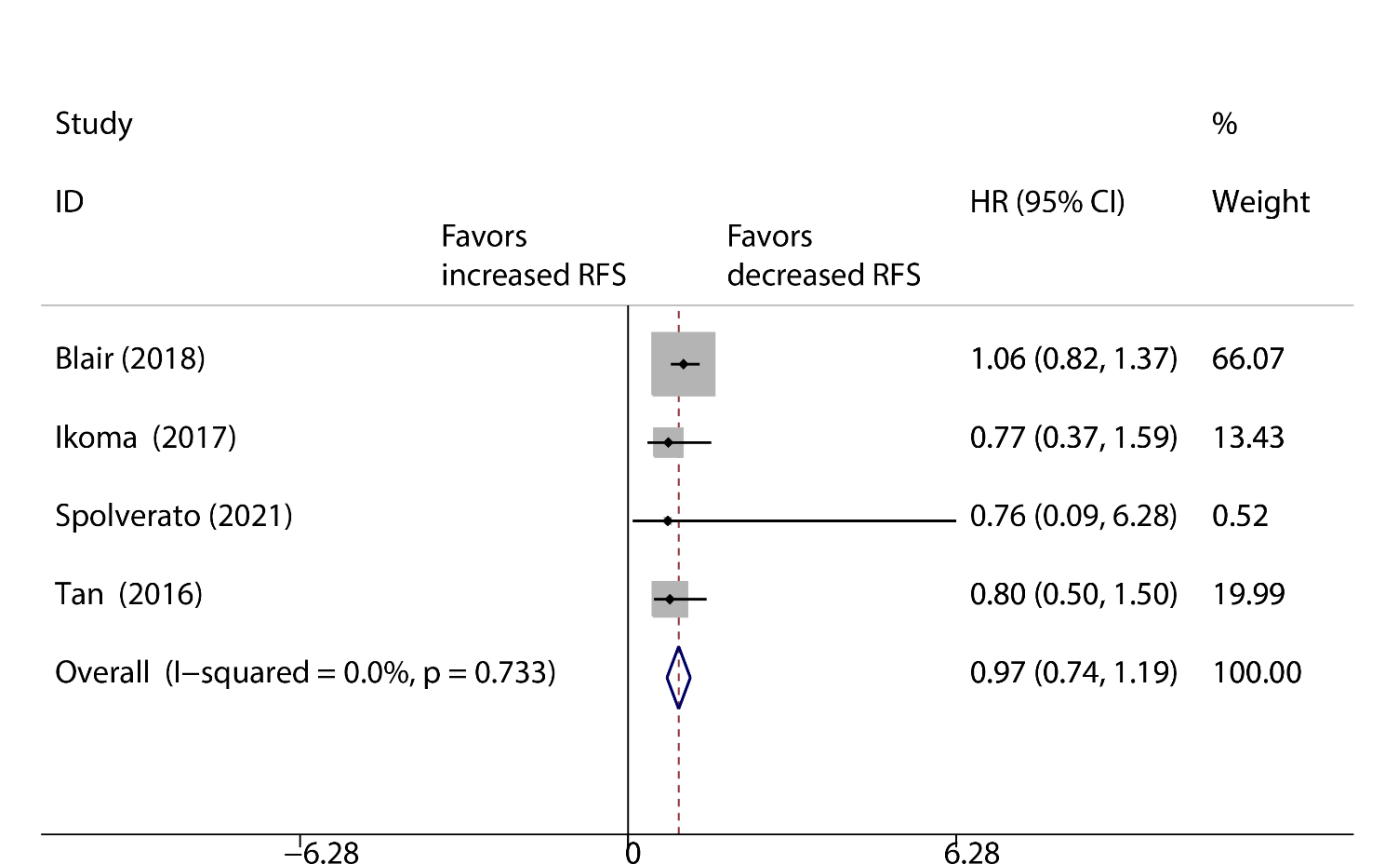
Pooled over-all survival of vascular resection versus tumour resection alone

There were four studies (959 patients) that reported RFS, and they were pooled in a fixed-effects model. The results showed no significant difference between the vascular resection group and the tumour resection alone group (HR: 0.97; 95% CI: 0.74–1.19; P = 0.945; Fig. 2), with no heterogeneity (I^2^ = 0%). We pooled the results of three studies (284 patients) that reported HRs for OS [10, 31, 32]. The results indicated no significant difference between the vascular resection group and the tumour resection alone group (HR: 1.01; 95% CI: 0.66–1.36; P = 0.774; Fig. 3), with no heterogeneity (I^2^ = 0%). Two trials reported no death related to vascular resection [10, 32]. Only one study compared major complication rates between the two groups [10], and vascular resection were burdened by a relatively higher rate of major complications (13/24, 54% vs. 5/24, 21%; OR: 2.60; 95% CI: 0.80–8.43; P = 0.111).

## Discussion

In this study, an aggressive surgical approach with vascular resection achieved acceptable rates of postoperative morbidity and mortality. The results of the meta-analysis demonstrated that the rates of postoperative morbidity and mortality were not significantly different between the extended resection group and tumour resection alone group. In addition, vascular resection achieved similar local recurrence or OS. For this systematic review and meta-analysis, we obtained all evidence so far published on the safety and long-term outcomes of vascular resection in RPS. To our best knowledge, our study is the first meta-analysis to comprehensively assess this issue.

Involvement of major vessels can be an indirect sign of the aggressiveness of RPSs [38], for which a multidisciplinary collaboration, including vascular surgeons, should be established [10, 34]. The decision for vascular reconstruction should be based on comprehensive evaluation of distant metastasis, tumour grade, organs involved, and the general condition of the patient. Furthermore, the feasibility and efficacy of vascular resection for RPS are yet to be determined. A single centre cohort study and a retrospective review of patients with intra-abdominal and RPSs showed that oncovascular surgery enables the radical resection required for good local control of RPSs and is associated with an acceptable level of complications peri-operatively and during follow-up [34]. The review of literature identified 37 articles with 110 patients, however, most of which were case reports. Besides, they did not perform a meta-analysis on the safety and long-term outcomes of vascular resection in RPS.

In this study, the results of included studies showed that most of the patients received total gross excision (R0/R1). We divided the studies into primary iliocaval leiomyosarcoma group and RPSs with vascular resection group. Postoperative early mortality rates were 0–20% and 0–8% in primary iliocaval leiomyosarcoma group and RPSs with vascular resection group, respectively. Major complication rates were 6–54% and 7%-54% in primary iliocaval leiomyosarcoma group and RPSs receiving vascular resection group, respectively. The 5-year OS rates were 33–78% and 50–69% in the primary iliocaval leiomyosarcoma group and RPSs with vascular resection group. The overall early postoperative mortality rate, major complication rate, and 5-year OS rate were similar between the primary iliocaval leiomyosarcoma group and RPSs with vascular resection group. The short-term and long-term outcomes were equivalent with the patients received extended resection including adjacent organs observed in some previous studies [39–41]. Thus, resection and reconstruction of the major vessels for en-bloc resection of RPS can be performed feasibly and safely.

Our data support the hypothesis that resection of major vessels should not be considered a contraindication to surgery in RPSs because the short-term and long-term clinical outcomes were similar between vascular resection group and tumour resection alone group. These results might be explained by the following reasons. Criteria mentioned in the included studies for vascular resection were encasement, involvement or vascular occlusion. Although the histological subtype, FNCLCC grade, tumour status were similar between vascular resection group and tumour resection alone group in propensity-matched analyses, status of involvement of major vessels were different between the two groups, which can be regarded as a more aggressive behavior [38]. Of note, resection of major vessels might improve surgical resection margins as compared with partial excision or no surgery. Our previous studies have indicated that surgical resection margins are correlated with long-term survival, and OS was higher in R0 resection than in R1 resection and in R1 resection than in R2 resection [42]. Thus, adjacent major vessels with evidence of direct invasion should be resected to avoid R2 resection.

With respect to the techniques for major vascular resection and reconstruction for RPS excision, different surgical strategies are needed for intraoperative situations. The most common major vessel involved in RPS is the IVC [43]. The methods of reconstruction of the IVC include blood vessel transplantation, repair, and ligation. Retroperitoneal tumours involving the IVC are usually divided into three segments: the infrarenal segment, the suprarenal infrahepatic segment, and the retrohepatic segment. Based on the results of included studies in this systematic review, most of the postoperative early mortality occurred in the patients with retroperitoneal tumours involving the retrohepatic segment of IVC. The main causes of death were hepatic failure and pulmonary embolism [17, 19, 20]. Primary sarcomas originating from the aorta are rare. Most of the arterial reconstructions in RPS patients were caused by secondary involvement, or encasement of the aorta wall or iliac arteries [43]. In cases of arterial resection, primary anastomosis is rarely feasible due to the length of the resection. Arterial reconstructions are usually performed using artificial vascular graft in an anatomic position. To improve the short-term results of surgical treatment, for RPSs with abundant blood supply from preoperative imaging examinations, especially those fed from the lumbar artery, middle sacral artery or internal iliac artery, tumour supply vessel embolisation was introduced in clinical practice. Studies have shown that early transarterial embolisation of the tumour supply vessels could significantly reduce intraoperative blood loss, operation time, and postoperative complication rate [44].

Major vessel injuries during oncological surgery can lead to serious bleeding, requiring massive transfusion [45]. To ensure the successful surgical resection of the advanced tumours, a multidisciplinary team, including vascular surgeons, is an essential component of the preoperative planning and co-operation with the postoperative management [46]. Locally advanced tumours involving adjacent major vessels require cooperation of the oncovascular surgeon as a multidisciplinary team member. Oncovascular surgery can be defined as cancer resection with concurrent ligation, or reconstruction of a major vascular structure [47]. Studies have continually supported the feasibility of surgical intervention with durable oncologic outcomes in various tumour pathologies with major vascular involvement, including pancreatic cancer, renal cell carcinoma, and cholangiocarcinoma [48]. Patients with RPS invading or intimately surrounding major vessels at the time of diagnosis have traditionally been regarded as a limitation for complete surgical resection and might result in an increased surgical morbidity. Advancements in vascular surgery techniques have resulted in the possibility of radical treatment being offered to RPS patients with major vessel involved which previously could not be operated on [34].

The strengths of our review include its comprehensive search and methodological robustness. We searched all available literature to exclude studies with overlapping cohorts and analysed large-scale studies. However, the present study also had some limitations. First, this review is based on non-confirmatory studies and secondary outcomes, and the histological subtype, FNCLCC grade, tumour status, and adjuvant therapy varied among the studies. Relevant data of some characteristics were lacking, possibly introducing bias. Second, there were an insufficient number of studies and patients included for meta-analysis, and subsequent subgroup analysis. Thus, the recommendations for these comparisons have a relatively weak power. A long-term prospective study in these areas is warranted. Finally, all trials included in the meta-analysis used an open-label design, which might introduce bias. However, assessment of the methodological quality of the included studies indicated that most studies had a low or medium risk of bias.

In conclusion, en-bloc resection with involved major vessels enables radical resection required for good local control of retroperitoneal sarcomas. Aggressive resection with involved major vessels can be performed safely with an acceptable level of complications and equivalent DFS and OS to that without vascular involvement. In patients with RPS, major blood vessels invasion would no longer be considered as technical non-resectability.

### Electronic supplementary material

Below is the link to the electronic supplementary material.

Supplementary Material 1

## Data Availability

All data generated or analysed during this study are included in this published article and its Additional files (all the studies that were included in this meta-analysis are included in Additional files).
